# A108 AUTOMATED DETECTION OF ILEOCECAL VALVE, APPENDICEAL ORIFICE, AND POLYP DURING COLONOSCOPY USING A DEEP LEARNING MODEL

**DOI:** 10.1093/jcag/gwac036.108

**Published:** 2023-03-07

**Authors:** M Taghiakbari, S Hamidi Ghalehjegh, E Jehanno, T Berthier, L di Jorio, A N Barkun, E Deslandres, S Bouchard, S Sidani, Y Bengio, D von Renteln

**Affiliations:** 1 University of Montreal, University of Montreal Hospital Research Center (CRCHUM); 2 Imagia Canexia Health Inc.; 3 McGill University, Division of Gastroenterology, McGill University Health Center, McGill University; 4 University of Montreal, Division of Gastroenterology, University of Montreal Hospital Center (CHUM); 5 University of Montreal, Montreal, Canada

## Abstract

**Background:**

Identification and photo-documentation of the ileocecal valve (ICV) and appendiceal orifice (AO) confirm completeness of colonoscopy examinations. We hypothesized that an artificial intelligence (AI)-empowered solution could help us automatically differentiate anatomical landmarks such as AO and ICV from polyps and normal colon mucosa.

**Purpose:**

We aimed to develop and test a deep convolutional neural network (DCNN) model that can automatically identify ICV and AO, and differentiate these landmarks from normal mucosa and colorectal polyps.

**Method:**

We prospectively collected annotated full-length colonoscopy videos of 318 patients undergoing outpatient colonoscopies. We created three non-overlapping training, validation, and test datasets with 25,444 unaltered frames extracted from the colonoscopy videos showing four landmarks/image classes (AO, ICV, normal mucosa, and polyps). For each landmark, we extracted an average of 30 frames for each time of its appearance. All the extracted frames were reviewed and annotated by a team of three clinicians. Using a quality assessment tool, the clinicians examined a total of 86,754 frames (7982 AO, 8374 ICV, 32,971 polyps, and 37,427 normal mucosa) and verified whether or not the frame contained one unique landmark. For this research, all frames were extracted from the white-light colonoscopies, and all narrow-band imaging frames were excluded. A DCNN classification model was developed, validated, and tested in separate datasets of images. The primary outcome was the proportion of patients in whom the AI model could identify both ICV and AO, and differentiate them from polyps and normal mucosa, with an accuracy of detecting both AO and ICV above a threshold of 40% (representing a value in which reliable identification of the landmarks can be assumed without increasing false-positive alerts).

**Result(s):**

We trained a DCNN AI model on 21,503 unaltered frames extracted from the recorded colonoscopy videos of 272 patients, and validated and tested the model on 1,924 (25 patients) and 2,017 (21 patients) unaltered frames, respectively. We applied a transfer learning technique to fine-tune the model parameters to the endoscopic images using a cross-entropy loss function and back-propagation algorithm. After training and validation, the DCNN model could identify both AO and ICV in 18 out of 21 patients (85.71%), if accuracies were above the threshold of 40%. The accuracy of the model for differentiating AO from normal mucosa, and ICV from normal mucosa were 86.37% (95% CI 84.06% to 88.45%), and 86.44% (95% CI 84.06% to 88.59%), respectively. Furthermore, the accuracy of the model for differentiating polyps from normal mucosa was 88.57% (95% CI 86.60% to 90.33%).

**Conclusion(s):**

The model can reliably distinguish these anatomical landmarks from normal mucosa and colorectal polyps. It can be implemented into automated colonoscopy report generation, photo-documentation, and quality auditing solutions to improve colonoscopy reporting quality.

**Please acknowledge all funding agencies by checking the applicable boxes below:**

Other

**Please indicate your source of funding;:**

MEDTEQ

**Disclosure of Interest:**

M. Taghiakbari: None Declared, S. Hamidi Ghalehjegh Employee of: Imagia Canexia Health Inc. , E. Jehanno Employee of: Imagia Canexia Health Inc. , T. Berthier Employee of: Imagia Canexia Health Inc. , L. di Jorio Employee of: Imagia Canexia Health Inc. , A. N. Barkun Grant / Research support from: co-awardee in funded research projects with Imagia Canexia Health Inc., Consultant of: Medtronic Inc. and A.I. VALI Inc, E. Deslandres: None Declared, S. Bouchard: None Declared, S. Sidani: None Declared, Y. Bengio: None Declared, D. von Renteln Grant / Research support from: ERBE, Ventage, Pendopharm, and Pentax, Consultant of: Boston Scientific and Pendopharm

